# Connections between Parental Phubbing and Electronic Media Use in Young Children: The Mediating Role of Parent–Child Conflict and Moderating Effect of Child Emotion Regulation

**DOI:** 10.3390/bs14020119

**Published:** 2024-02-06

**Authors:** Xiaocen Liu, Shuliang Geng, Tong Lei, Yan Cheng, Hui Yu

**Affiliations:** College of Preschool Education, Capital Normal University, Beijing 100048, China; 2223102004@cnu.edu.cn (S.G.); 2223102026@cnu.edu.cn (T.L.); 2223102032@cnu.edu.cn (Y.C.); 2223102058@cnu.edu.cn (H.Y.)

**Keywords:** young children, phubbing, electronic media, parent–child conflict, emotion regulation

## Abstract

In this digital age, where parental attention is often diverted by digital engagement, the phenomenon of “parental phubbing,” defined as parents ignoring their children in favor of mobile devices, is scrutinized for its potential impact on child development. This study, utilizing questionnaire data from 612 parents and Structural Equation Modeling (SEM) with moderated mediation, examines the potential association between parental phubbing and young children’s electronic media use. The findings revealed a correlation between parental phubbing and increased electronic media use in children. Parent–child conflict, informed by instances of parental phubbing, was identified as a partial mediator in this relation. Notably, children’s emotion regulation emerged as a moderating factor, with adept regulation linked to reduced adverse effects of parental phubbing and improved relational harmony. These findings underscore the importance of parental awareness of their digital behaviors and the benefits of fostering robust parent–child relationships and supporting children’s emotional regulation to nurture well-adjusted “digital citizens” in the contemporary media landscape.

## 1. Introduction

In today’s digital age, especially in the aftermath of the coronavirus disease 2019 (COVID-19) pandemic, the behavior of parental phubbing has become alarmingly pervasive, raising profound concerns about its detrimental impact on children’s psychological and emotional well-being. Parental phubbing refers to parents excessively diverting their attention to electronic devices and neglecting face-to-face interactions with their children [1,2,3,4]. This behavior has become seamlessly integrated into our daily lives as information technology continues to advance at an unprecedented rate [5]. A study by Blackman found that parents, on average, dedicate between 0.5–7.5 h to mobile device use daily, with up to 5 h spent on this behavior in their children’s presence [6]. Moreover, 65% of mothers have acknowledged technology as a source of distraction during critical parent–child interactions [7]. The post-pandemic era brought with it unique challenges: as measures like self-quarantine were enforced to curb the spread of COVID-19, individuals spent more time at home, inadvertently leading to increased exposure to electronic devices [8]. Trott et al. conducted a systematic review and meta-analysis, identifying a significant surge in adult screen time during the pandemic, with a daily increase averaging 1 h more than pre-pandemic levels [9]. Similarly, a study by Alheneidi et al. during the COVID-19 lockdown period reported that more than half of the 593 participants spent at least 6 h per day online, with 31.5% exceeding 8 h. The ramifications of this behavior are alarming [10]. Overexposure to parental phubbing can induce feelings of neglect in children and contribute to developing insecure attachments [11], potentially leading to aversions to intimate relationships later in life [2]. A telling observation by Vanden Abeele et al. highlighted that parents engrossed in mobile phones were five times less likely to attend to their children’s attention-seeking gestures than when not using phones [12]. Given the gravity of these findings, there is an imperative need for scholars and professionals to delve deeper into this phenomenon, aiming to devise strategies to mitigate its adverse effects.

As the post-pandemic era unfolds, children’s electronic media use, which encompasses not only traditional platforms like television and DVDs but also extends to mobile phones, computers, tablets, and various forms of interactive and streaming services [13], has seen a significant uptick [9], reflecting a broader societal shift toward digital media. This epoch, characterized by a move toward digital platforms for educational and recreational purposes, has intensified the prevalence of electronic media use among children. As delineated by Geng et al., the inception of electronic media interaction among children occurs at a tender average age of 2.45 years, underscoring a trend where younger cohorts are progressively engaging with such media [14]. Bar a negligible fraction, nearly all children in the studied cohorts had prior exposure to electronic media, accentuating its ingrained nature in their daily lives. The consequences of children’s extensive electronic media exposure are far-reaching, affecting multiple dimensions of child development. Early and frequent engagement with electronic media has been linked to impairments in executive function [15]. This pervasive interaction with electronic media has also been linked to the emergence of internalizing problems, with symptoms such as depression and anxiety [14,16,17,18]. Additionally, this exposure can adversely affect social development in later life stages [19,20,21]. A specific aspect of electronic media use, problematic video gaming, has been associated with neurophysiological changes in minors, particularly in the prefrontal cortex and striatum, which may compromise cognitive control [22]. Importantly, these effects are more common in younger children [23]. In light of these findings, there is an urgent need to emphasize the importance of informed parental guidance. Such guidance is essential for creating a digital environment that supports the holistic development of children in an evolving digital world.

Building upon the abovementioned issues, the expanding realm of digital engagement underscores a pressing need to investigate the interconnections among parental digital behaviors, child media usage, and the resultant dynamics within parent–child interactions. As expounded in the preceding discourse, the ubiquity of parental phubbing and children’s expansive access to electronic media, particularly in the post-pandemic era, not only epitomizes the digital zeitgeist but also beckons forward a comprehensive elucidation of its ramifications on early developmental realms. The present study embarks on a relatively uncharted terrain to decipher the complex interplay of parental phubbing, parent–child conflict, children’s electronic media use, and the mitigative potential of children’s emotion regulation within this framework. This endeavor transcends a mere academic exercise, manifesting as a pertinent inquiry within the contemporary digital familial ecosystem, wherein digital interactions are increasingly enmeshed in the tapestry of the parent–child relationship [7]. The insights derived from this investigation are envisaged to cultivate a nuanced comprehension of the digital paradigm within familial contexts, laying the groundwork for informed interventions to foster conducive parent–child dynamics amidst the digital age vicissitudes. The dynamics mentioned above highlight the necessity of a detailed examination of the existing literature so as to contextualize the current inquiry within the broader scholarly discourse and identify the gaps this study aims to fill.

### 1.1. Parental Phubbing and Children’s Electronic Media Use

Numerous studies have demonstrated that parental phubbing is associated with elevated levels of electronic media use among children; for instance, Wang et al. found that parental phubbing contributed to increased electronic media engagement in young children [24]. Zhou et al. similarly uncovered a significant positive correlation between parental phubbing and children’s Internet gaming addiction in a study involving 1021 Chinese children and their parents [2]. Further corroborating these findings, Zhao et al. conducted research during the COVID-19 pandemic that confirmed this positive association [8]. Finally, a review by McDaniel on the effects of parental phubbing on child development complemented these findings, indicating that children who perceive their parents as consistently engaged with electronic devices tend to use their phones more and are more likely to experience depression, thus further emphasizing the potential consequences of parental phubbing on children’s electronic media use [25].

Bandura’s Social Learning Theory is a foundational framework for exploring the relation between parental phubbing and children’s electronic media use [26]. This theory emphasizes observational learning as the primary mechanism through which children adopt behaviors, suggesting that children not only witness but also internalize the norms and values associated with electronic device usage. The concept of “vicarious reinforcement” adds depth to this theory, positing that children are likelier to mimic behaviors that they perceive as rewarding for their parents. Complementing this, Attachment Theory, initially developed by Bowlby and later expanded by Ainsworth, introduces an emotional perspective [27]. This theory posits that disruptions in the emotional bonds between caregivers and children, such as those caused by parental phubbing, can lead to emotional and behavioral challenges, prompting children to seek solace in electronic media. Adding a cognitive layer to this discussion, the Technology Acceptance Model posits that technology adoption is influenced by two primary factors: perceived ease of use and usefulness [28]. In the context of parental phubbing, when children see their parents frequently interacting with electronic devices, they may view these devices as both user-friendly and beneficial, increasing their propensity to engage with electronic media. Taken together, Social Learning Theory, Attachment Theory, and the Technology Acceptance Model offer a comprehensive theoretical framework that elucidates the various mechanisms—from behavioral imitation and emotional coping strategies to cognitive attitudes—that influence the impact of parental phubbing on children’s electronic media use.

Building upon the above theories, we propose Hypothesis H1: Parental phubbing and electronic media use among young children are positively correlated. In other words, parental engagement with electronic devices in the presence of their children is associated with increased electronic media use by the children themselves.

### 1.2. The Mediating Role of Parent–Child Conflict

Parent–child conflict facilitates the development of negative parent–child relationships—specifically, the continuance of psychological conflicts or external opposing behaviors between parents and children due to cognitive, emotional, and behavioral incompatibilities [29,30,31,32]. Attachment theory posits that a positive attachment bond with caregivers fosters cognitive and emotional growth, while avoidant attachment may lead to social adjustment difficulties [27]. The effects of parental behaviors inevitably transfer to children’s feelings through interactions and are expressed through the latter’s external behaviors. Individuals who develop insecure attachments often struggle to trust their parents, generalizing this feeling to others [33]. Such generalized feelings of mistrust can lead to a tendency to withdraw from interpersonal interactions, resulting in social adjustment problems [34,35]. Electronic media’s anonymity and other features can dissolve the low trust experienced by avoidant-attached individuals, subsequently increasing their usage of electronic media [2]. Based on this theory, parent–child conflict may mediate the association between family environment (e.g., parental phubbing) and children’s use of electronic media.

Phubbing behavior by parents can lead to conflict with their children. During interactions, frequent parental phubbing behaviors may result in lower-quality communication, neglect of the children’s needs, and the formation of emotional distance [4,11,17,36,37]. Technology interference between parents and children indicates that high levels of electronic media exposure can disrupt communication with other family members, hindering the formation of a positive parent–child relationship [7]. Radesky et al. also underlined that parents addicted to electronic media often perceive their children’s interaction needs as intrusive [38]. Consequently, they are more likely to respond hostilely and exhibit more apathetic behaviors. Furthermore, Informal Social Control theory emphasizes that an indifferent, distant family atmosphere may amplify an individual’s negative emotions [39]. Therefore, parents’ high-frequency phubbing may significantly trigger parent–child conflict.

Parent–child conflict predicts children’s electronic media use [2,40,41,42]. Studies have shown that a close parent–child relationship, characterized by emotional warmth, can reduce a child’s need to seek a sense of belonging in the virtual world [10,29]. In contrast, poor parent–child relationships can lead to feelings of parental rejection and social exclusion [43]. Individuals in such relationships may avoid close relationships and seek to escape loneliness through virtual worlds [10,44]. Furthermore, individuals with insecure attachments often struggle to trust others and may avoid real-world contact [34]. Previous research has demonstrated that parent–child conflict, as a proximal family factor, significantly predicts children’s electronic media addiction [2,22].

In light of this, we propose Hypothesis H2: Parent–child conflict mediates the association between parental phubbing and young children’s use of electronic media. Specifically, parental phubbing may be associated with heightened conflicts, which coincides with a tendency for children to engage more with electronic media for comfort.

### 1.3. The Moderating Role of Children’s Emotion Regulation

In the pathway from parental phubbing through parent–child conflict to young children’s electronic media use, children’s emotion regulation may be a moderating variable. Numerous scholars have argued that, aside from home environment factors such as parental behavior, children’s factors, including emotion regulation, are significantly associated with parent–child relationships [45,46]. Strelau posited that intra-individual variability significantly impacts sensory stimuli, thereby causing inter-individual differences in the perception of stimulus intensity [47]. In simpler terms, different individuals perceive the same stimulus with varying intensity levels due to dissimilarities in their regulatory mechanisms. The Integrated Model of Emotion Processes and Cognition in Social Information Processing also indicates that the individual’s emotional processes influence an individual’s processing of social information in a particular situation [48].

Emotion regulation refers to an individual’s dynamic processing of the occurrence, experience, and expression of their emotions [49,50,51,52]. It plays a crucial role in how individuals process and respond to harmful stimuli, such as parental phubbing. Individuals with emotional difficulties tend to be more affected by adverse events, which can amplify their negative impact [53,54]. This heightened sensitivity can lead to more challenges and difficulties in parenting, potentially increasing the frequency of harsh parenting practices and punishment [49,55]. Such practices can intensify conflicts between parent and child, subsequently deteriorating the parent–child relationship [56]. In contrast, individuals with positive emotion regulation may experience more favorable affective feelings and employ more adaptive strategies to reduce stimulus intensity in the face of negative stimuli [57,58], contributing to secure parent–child attachments [59].

Given the potential of emotion regulation to act as a protective factor, shaping children’s perceptions of parental phubbing in a way that fosters positive attributions and mitigates the detrimental impact on parent–child relationships, we put forth Hypothesis H3: Young children’s emotion regulation may serve as a moderating variable in the relation between parental phubbing and parent–child conflict.

### 1.4. The Present Study

Contemporary research in child development has increasingly acknowledged the influence of parental phubbing, but there remains a substantial gap in understanding its intricate connections with electronic media use in young children. While some studies have considered parent–child conflict in the context of parental phubbing, few have delved into its role as a mediating factor linking parental phubbing to young children’s use of electronic media. This oversight is particularly crucial in the digital age, where parental phubbing may often lead to parent–child conflict, affecting children’s media behavior.

In addition to these factors, it is essential to consider the role of children’s age in their electronic media use and emotion-regulation capabilities. Research, including studies by Poulain et al., Camargo and Orozco, and Duch et al., has shown a relation between age and media use in young children [60,61,62]. Furthermore, the development of emotion regulation abilities in children, influenced by neurological development [63], cognitive maturation [64], and social experiences [65], enhances with age. Accordingly, our model includes age as a control variable to account for its potential association.

**Figure 1:**
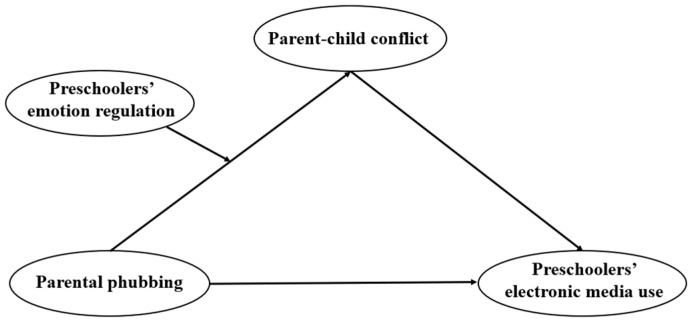
The moderated mediation model. Age is included as a control variable in the model.

In response to this, the study shifts the focus from perceiving children as merely passive recipients within this dynamic to recognizing their active role, primarily through the lens of how their emotional-regulation capacities may act as a moderating variable in the relation between parental phubbing and parent–child conflict, and how this, in turn, influences their use of electronic media. Through this approach, by proposing a moderated mediation model (Figure 1), the present study aims to fill the existing research gaps and provide a more holistic understanding of child development in the context of digital media interactions.

The specific objectives of this study are to (a) elucidate the relations among the variables, rigorously examining the associations among parental phubbing, parent–child conflict, children’s emotion regulation, and electronic media use; (b) verify the mediating role of parent–child conflict in the pathway from parental phubbing to children’s electronic media use; and (c) assess the moderating effect of children’s emotion regulation in the relation between parental phubbing and parent–child conflict and subsequently explore its association with children’s electronic media use. Through this investigation, we seek to provide evidence and support for a more nuanced understanding of the role of parental phubbing and the development of interventions targeting young children’s electronic media use.

## 2. Materials and Methods

### 2.1. Participants and Procedure

In this study, we adopted convenience sampling, with educators in public, private, and other kindergartens throughout Beijing’s 16 districts assisting in disseminating anonymous questionnaires to parents who have daily interactions with their children. The Institutional Review Board granted ethical approval, and informed consent was obtained from all participants. We ensured participants clearly understood key study terms, like “electronic media use”, to improve the accuracy of their responses.

From the initial pool, we excluded 68 questionnaires due to non-residency, incompleteness, or inconsistencies, resulting in a sample size of 612 parents. This sample included 171 fathers (27.94%) with an average age of 33.56 years (*SD* = 4.88) and 441 mothers (72.06%) with an average age of 31.82 years (*SD* = 4.47). The children’s ages ranged from 1.09 to 6.93 years, averaging 4.68 years (*SD* = 1.18), with 299 boys (48.86%) and 313 girls (51.14%). There were 123 siblings (20.10%) and 489 only children (79.90%), with 465 (75.98%) from urban and 147 (24.02%) from non-urban areas.

### 2.2. Measures

#### 2.2.1. Parental Phubbing

Parental phubbing was assessed using the Parental Phubbing Scale, which was initially developed by Roberts and David [66] and later revised by Zhao et al. [8]. This scale features a unidimensional structure and consists of nine items, such as “I glance at my cell phone when talking to my child”. Participants rated their phubbing behavior using a 5-point Likert scale, where 1 point signifies “never”, and 5 points represent “all the time.” Higher scores on the scale indicate a more frequent occurrence of phubbing behavior by parents. In this study, the Cronbach’s alpha coefficient for the scale was 0.78.

#### 2.2.2. Parent–Child Conflict

The Child–Parent Relationship Scale formulated by Pianta [30] and revised by Zhang [31] was adopted in this study. The scale includes 22 items and is divided into the closeness and conflict subscales, which capture different facets of the parent–child relationship. This study used the conflict subscale containing 12 items (e.g., “My child easily becomes angry at me”). All items are rated on a 5-point Likert scale from definitely does not apply to definitely applies. Higher scores on the conflict subscale indicate the existence of more intense conflict between parents and children. In this study, the Cronbach’s alpha coefficient for the Child–Parent Relationship Scale was 0.88, and the Cronbach’s alpha coefficient for the conflict subscale was 0.92, indicating that the conflict subscale has high internal consistency.

#### 2.2.3. Emotion Regulation

This study used the Emotion Regulation Checklist, which was initially formulated by Shields and Cicchetti [50] and subsequently revised by Liu et al. [67], to evaluate emotion regulation. The checklist covers two dimensions, emotional instability and emotion regulation, and includes a total of 21 items. For this study, we focused on the emotion regulation subscale, which consists of eight items (e.g., “My child is empathic toward others”). Items are rated on a 4-point Likert scale, with higher scores on the emotion regulation subscale indicating better development of children’s emotion regulation abilities. The Cronbach’s alpha coefficient for this study’s overall Emotion Regulation Checklist was 0.78, while the coefficient for the emotion regulation subscale was 0.74, suggesting acceptable internal consistency.

#### 2.2.4. Electronic Media Use

To assess electronic media use, this research employed a questionnaire initially crafted by Huang et al. [68] and subsequently refined to include 14 items by Geng et al. [14] through confirmatory factor analysis. The items, such as “My child spends less time playing outdoors because of the use of electronic media”, span four dimensions: electronic media time management, interpersonal and health conditions caused by electronic media use, life conflicts arising from electronic media use, and emotional experiences related to electronic media use. Participants rated each item on a 5-point Likert scale, where 1 point signifies “strongly disagree” and 5 points denote “strongly agree”. A higher score suggests a more severe level of electronic media engagement. The Cronbach’s alpha coefficient for this questionnaire in the current study was 0.94, indicating excellent internal consistency.

### 2.3. Data Analysis

We used SPSS version 23.0 (IBM Corporation, Armonk, NY, USA) for initial data input, collation, and preliminary analyses. The normality of the data distribution was assessed using Q–Q plots [69], where the scatter points closely aligned with the diagonal, confirming the assumption of data normality. We employed Harman’s single-factor test to address the potential common method bias issue. Variable scores were computed, and relations among key variables, such as parental phubbing, parent–child conflict, emotion regulation, and electronic media use, were explored using Pearson’s correlation method. Following these preliminary analyses, we constructed and validated structural equation models using AMOS version 26.0 (IBM Corporation). This advanced analytical phase sought to elucidate the relation between parental phubbing and young children’s electronic media use, accounting for the mediating role of parent–child conflict and the moderating role of emotion regulation.

## 3. Results

### 3.1. Common Method Bias

To mitigate the risk of common method bias, we implemented anonymous data collection and incorporated reverse-scoring of items in the questionnaire design. Despite relying on self-reported data, additional steps were taken to assess the potential for common method bias using Harman’s single-factor test. In line with the guidelines set forth by Tang and Wen [70] for research conducted in China, the variance explained by a single factor should not exceed 40%. Our study employed exploratory factor analysis to assist with Harman’s single-factor test. The results indicated that the first factor accounted for 29.68% of the variance, falling below the 40% threshold. Therefore, we concluded that common method bias was not a significant concern in this study.

### 3.2. Preliminary Analysis

Initial analyses indicated that the mean score for fathers’ phubbing behavior was 2.71 points (*SD* = 0.58), while for mothers, it was 2.64 points (*SD* = 0.65). An independent-samples *t* test revealed no significant difference between the phubbing behaviors of fathers and mothers (*t* (342.42) = 1.32, *p* = 0.19). After controlling for the children’s age, further investigation using multiple-group analysis in AMOS version 26.0 assessed whether there was a differential association between fathers’ and mothers’ phubbing and children’s electronic media use. The results indicated that both fathers’ (β = 0.34, *p* < 0.001) and mothers’ (β = 0.49, *p* < 0.001) phubbing was significantly and positively associated with children’s electronic media use, with no significant difference between the two groups (*p* = 0.49). Additionally, the mean score for electronic media use was 2.34 points (*SD* = 0.76) for boys and 2.31 points (*SD* = 0.78) for girls. An independent-samples *t* test found no significant differences in electronic media use between boys and girls (*t* (610) = 0.35, *p* = 0.72). Consequently, subsequent analyses did not differentiate between the sexes of children or the phubbing behavior of fathers and mothers.

### 3.3. Descriptive Statistics and Correlation Analysis

Table 1 presents the descriptive statistics and correlations for the primary variables under investigation. Parental phubbing was positively associated with parent–child conflict and children’s electronic media use. In contrast, the association between parental phubbing and children’s emotion regulation was not statistically significant. Parent–child conflict negatively correlated with children’s emotion regulation and positively correlated with electronic media use. Emotion regulation and electronic media use were inversely related, albeit with weaker relations. Age was positively correlated with emotion regulation and electronic media use, which were comparatively weaker. Consequently, children’s age was controlled for in subsequent analyses.

### 3.4. Moderated Mediation Effect Test

We used AMOS version 26.0 to construct a Structural Equation Model (SEM) to test the hypothesized model. The maximum likelihood method was employed, with 5000 resamples and a 95% confidence interval. Given that the measurement instruments in this study included both multidimensional scales (Electronic Media Use Questionnaire) and unidimensional scales (Parental Phubbing Scale, Parent–Child Conflict Subscale, and Emotion Regulation Subscale), we followed the item-parceling recommendations of Little et al. [71]: specifically, we applied isolated parceling for the multidimensional Electronic Media Use Questionnaire, wherein each subscale was condensed into a single indicator. For the unidimensional scales, we employed the factorial algorithm for item parceling, as suggested by Rogers and Schmitt [72], to improve model fit.

Using structural equation analysis to account for children’s age as a control variable, we examined the relation between parental phubbing and children’s electronic media use. The model demonstrated excellent fit, as evidenced by the following indices: χ^2^/df = 2.06, RMSEA = 0.04, GFI = 0.98, NFI = 0.99, RFI = 0.98, IFI = 0.99, TLI = 0.99, and CFI = 0.99. The path linking parental phubbing to children’s electronic media use was statistically significant and positive (β = 0.45, *p* < 0.001, 95% CI [0.37, 0.52]). In simpler terms, higher levels of parental phubbing were associated with greater electronic media use by children.

In order to explore the mediating role of parent–child conflict in the relation between parental phubbing and children’s electronic media use, we incorporated this variable into our initial structural equation model. The model fit indices were as follows: χ^2^/df = 2.83, RMSEA = 0.05, GFI = 0.97, NFI = 0.97, RFI = 0.96, IFI = 0.98, TLI = 0.98, and CFI = 0.98. We observed the existence of a significant positive path from parental phubbing to parent–child conflict (β = 0.53, *p* < 0.001, 95% CI [0.46, 0.60]) as well as one from parent–child conflict to children’s electronic media use (β = 0.49, *p* < 0.001, 95% CI [0.39, 0.58]). Bootstrap tests confirmed a significant mediating effect (*ab* = 0.26, *p* < 0.001, 95% CI [0.20, 0.33]). These findings indicate that parent–child conflict partially mediates the relation between parental phubbing and children’s electronic media use, accounting for 57.72% of the total effect. In simpler terms, a higher frequency of parental phubbing was associated with more reported parent–child conflicts, which, in turn, corresponded with increased electronic media use among children.

**Figure 2:**
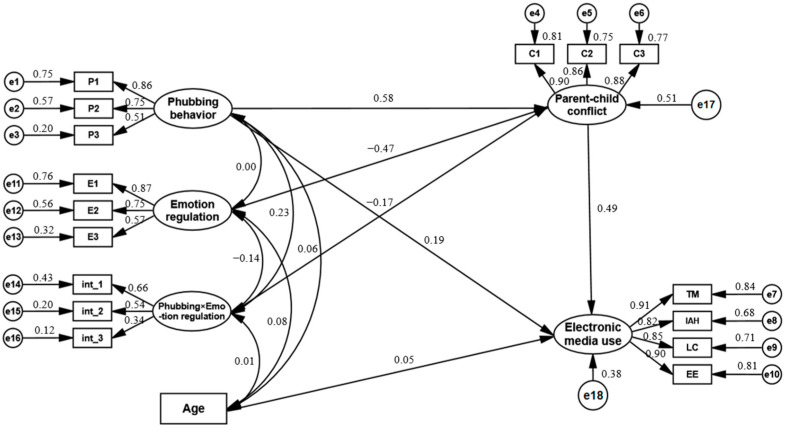
Mediating effect of parent–child conflict on the relation between parental phubbing and children’s electronic media use and the moderating role of children’s emotion regulation.

We extended the Structural Equation Model to examine further the moderating role of children’s emotion regulation in the first half of the mediated model pathway. Emotion regulation, conceptualized as a moderator in our study, was integrated into the model along with interaction terms between emotion regulation and parental phubbing (post-centering). These interaction terms—int_1, int_2, and int_3—were constructed based on the factor loadings of the indicators for parental phubbing and emotion regulation, following the paired product method. It is critical to note that in the SEM, emotion regulation does not demonstrate a direct correlation with parental phubbing, which serves as the independent variable. The absence of a direct effect aligns with the theoretical understanding that moderation involves an interaction effect rather than a straightforward association. The model fit indices were as follows: χ^2^/df = 2.90, RMSEA = 0.06, GFI = 0.95, NFI = 0.94, RFI = 0.92, IFI = 0.96, TLI = 0.95, and CFI = 0.96, indicating an excellent fit. Bootstrap tests revealed a significant negative association from the interaction term between parental phubbing and children’s emotion regulation to parent–child conflict (β = −0.17, *p* < 0.01, 95% CI [−0.32, −0.07]), which suggests that the strength of the relation between parental phubbing and parent–child conflict is contingent upon the level of the child’s emotion regulation. A significant path from emotion regulation to parent–child conflict was also observed (β = −0.47, *p* < 0.001, 95% CI [−0.57, −0.38]), underscoring the role of children’s emotion regulation as a moderating variable in this dynamic. These findings, elaborated in Figure 2, highlight the significant prediction of a parent–child conflict by the interaction between parental phubbing and emotion regulation, with adept regulation specifically reducing the adverse effects of parental phubbing on the parent–child relationship and enhancing relational harmony.

**Figure 3:**
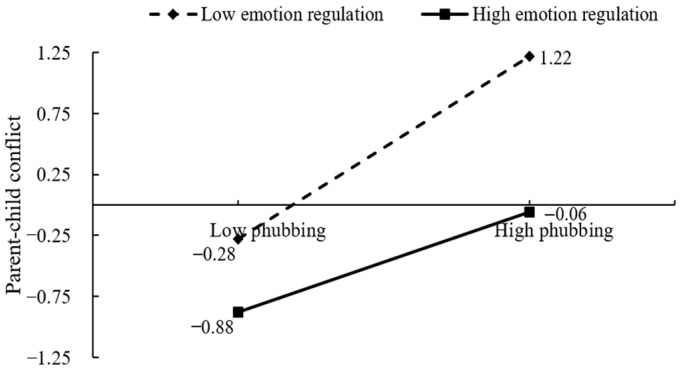
Simple slope analysis of the interaction between parental phubbing and children’s emotion regulation in parent–child conflict.

A simple slope analysis was employed to elucidate the moderating effect of children’s emotion regulation on the relation between parental phubbing and parent–child conflict, as delineated in Figure 3. For children demonstrating lower emotional regulation (M − 1SD), a marked positive correlation between parental phubbing and parent–child conflict was observed (β = 0.74, *p* < 0.001, 95% CI [0.58, 0.97]). Similarly, among those with higher emotional regulation (M + 1SD), this positive relation persisted but was less pronounced (β = 0.41, *p* < 0.001, 95% CI [0.29, 0.51]). The findings underscore the increased susceptibility of children with low emotional regulation to intensified parent–child conflict in the wake of escalating parental phubbing. In contrast, a higher level of emotion regulation in children was associated with a reduced association between parental phubbing and negative parent–child interactions.

## 4. Discussion

### 4.1. Parental Phubbing and Children’s Electronic Media Use

The study’s findings indicate a significant association between parental phubbing and higher levels of electronic media use in young children. The data show that higher levels of media use among children are associated with similar amounts of phubbing behavior from both mothers and fathers. This result suggests that the act of phubbing itself, regardless of the parent’s sex, has a substantial role in the media-engagement levels of young children.

Diving deeper into theoretical explanations, we can draw upon several frameworks. Social Learning Theory suggests that children learn behaviors through observation and imitation, implying that children may adopt media use habits directly from their phubbing parents [26]. Attachment Theory posits that disruptions in emotional bonding due to parental phubbing could prompt children to seek connection through alternative means [27], such as electronic media. Ecological Systems Theory contextualizes these interactions within the broader environment that shapes child development, emphasizing the direct influence of family behaviors on a child’s growth [73]. The Technology Acceptance Model offers a cognitive perspective, arguing that children perceive electronic devices as useful and easy to use, particularly when they see their parents frequently engaged with such technology [28].

In light of the theoretical frameworks, our analysis suggests that maternal and paternal phubbing behaviors are similarly associated with young children’s media usage patterns. This observation underscores the importance of involving both parents in interventions to cultivate healthy media habits among children, reflecting their joint role within family dynamics.

### 4.2. Mediation of Parent–Child Conflict

Our study found that parent–child conflict acted as a mediating factor in the relation between parental phubbing and children’s engagement with electronic media. Specifically, we observed that higher parental engagement with mobile devices correlates with more frequent parent–child conflicts. These conflicts are associated with a tendency for children to turn to electronic devices, possibly seeking solace or distraction. This pattern of mediation aligns with our research hypothesis. It illustrates the complex dynamics between parental digital habits, the quality of their interaction with their children, and the children’s subsequent media usage.

The association between parental phubbing and parent–child conflict can be interpreted within the frameworks of Attachment Theory, Informal Social Control Theory, and the Substitution Hypothesis. The frequent engagement of parents with their digital devices disrupts the emotional communication necessary for a secure parent–child relationship, as Shen et al. [74] indicated. This disruption often leads to feelings of neglect in children [25], as Wang et al. observed, with children responding by exhibiting escalated behaviors, such as shouting or crying, to gain parental attention [1]. Attachment Theory suggests that this emotional neglect strains the secure bond essential for a child’s healthy emotional development, potentially manifesting in increased conflict [27]. Simultaneously, Informal Social Control theory proposes that the family environment shapes individuals’ behaviors and emotional responses. The lack of parental responsiveness and patience, as noted by McDaniel and Coyne [7] and Vanden Abeele et al. [12], can lead to harsher disciplinary styles [49], exacerbating parent–child conflicts [25]. Furthermore, the Substitution Hypothesis posits that parents’ time on digital devices can replace valuable interaction time with their children, reducing cohesion and increasing parent–child conflict [75,76]. The findings from Shen et al. [74], which suggest an association between parental phubbing, reduced emotional warmth, and a detached family atmosphere, align with our observations of the mediating role of parent–child conflict.

Through the lens of Parental Acceptance–Rejection Theory and Compensatory Satisfaction Theory, we understand the connection between increased parent–child conflict and children’s heightened use of electronic media. Parental Acceptance–Rejection Theory highlights how emotional rejection within the family, common in conflictual relationships, drives children to seek connection and validation through electronic media [77]. This search for emotional connection is exacerbated in children who, due to insecure attachment relationships, often struggle with trust and intimacy, leading to feelings of loneliness [33,78,79,80]. Compensatory Satisfaction Theory posits that children turn to virtual environments to fulfill these unmet psychological needs, especially when family interactions are strained [81]. Children may increase their electronic media use to alleviate loneliness through anonymous virtual interactions [10,82]. This dynamic suggests a higher risk of electronic media addiction as parent–child conflict escalates, underscoring the need for fostering positive family relationships to mitigate this risk.

### 4.3. Moderation of Emotion Regulation

Our investigation has confirmed that children’s emotion regulation significantly moderates the effect of parental phubbing on parent–child conflict, providing support for Hypothesis H3. This result suggests that the capacity for emotion regulation might be associated with how children respond to parental phubbing, which could be related to the dynamics of parent–child conflict. This regulatory ability is crucial in shaping children’s perceptions and responses to parental engagement with digital devices, signifying their proactive role within the family unit. Moreover, the ability to regulate emotions not only tempers immediate reactions to parental phubbing but also informs broader behavioral patterns, including electronic media use.

Emotion regulation in children notably moderates the association between parental phubbing and parent–child conflict, aligning with Strelau’s perspective on the role of internal regulatory mechanisms in shaping responses to stimuli [47]. Children with more mature emotion regulation abilities could mitigate the adverse effects of parental phubbing by employing adaptive strategies to manage the potential for conflict [48,49,57,58]. Conversely, less developed emotion regulation in children magnifies the negative repercussions, exacerbating parent–child conflict, as Banks et al. [53] and Zhou et al. [2] demonstrated. This dynamic interplay underscores the bi-directionality of parent–child relationships [83], where children’s responses to parental behavior, influenced by their emotion regulation capabilities, significantly shape the family environment [45]. The findings corroborate the notion that proficient emotion regulation enables children to modify their emotional responses, creating a buffer against negative stimuli like parental phubbing, bolstering the resilience of the parent–child bond.

### 4.4. Limitations and Future Directions

Our study provides valuable insights into child development in the digital era. While the questionnaire method offers significant findings, it does not enable us to infer causality. For instance, it is possible that children’s electronic media use could influence parental phubbing. Experimental study designs could prove beneficial for future research aimed at exploring causal relations. Such methodologies might provide more precise insights into the effects of various variables within the context of parental phubbing and child development.

The study’s reliance on parental self-reports presents another limitation, as these self-reported measures may reflect biases like social desirability or inaccuracies in recall. Incorporating more objective measures, such as actual screen time logs or observational techniques, would likely yield more reliable and detailed data. Additionally, extending this research to include children’s perspectives would offer a richer understanding.

While focusing on the Beijing population has its merits, broadening the research scope to include diverse socio-economic and cultural contexts would enhance the generalizability and applicability of our findings. Considering the roles of other family members and external factors like peer relationships and school environments may also yield a more holistic view of the variables influencing children’s media use.

Lastly, given the rapid evolution of digital media, ongoing research is essential to keep abreast of its ever-changing impacts on family dynamics and child development. As the digital engagement landscape transforms, understanding its effects remains a moving target, necessitating continual study and adaptation in research approaches.

### 4.5. Implications

The findings of our study contribute to a better understanding of early childhood development in the context of the digital era. While much of the existing research in electronic media use has traditionally focused on older children and adults, our study provides insights into the dynamics within younger children’s environments. We explore the interplay of family factors such as parental phubbing and parent–child conflict, along with individual factors like emotion regulation, concerning young children’s media usage. This investigation enriches the application of Ecological Systems Theory and Attachment Theory in understanding the impact of digital media on early childhood development. These insights are valuable for developing informed approaches to parenting and supporting the holistic development of children in today’s digitally influenced environment.

Informed by these theoretical insights, this research endeavors to provide crucial practical implications for fostering child development in an increasingly digitalized world. This study underscores the importance of parental awareness regarding their digital habits, particularly phubbing, and its association with children’s emotional and cognitive development. This awareness is critical to creating an environment that supports healthy development. Furthermore, our study highlights the association between children’s emotion regulation skills and their ability to navigate the challenges of parental digital engagement. This finding points to the potential value of supporting the development of emotion regulation skills in children, which may be related to their experiences in parent–child relationships in the context of digital engagement. Additionally, it suggests using technological solutions like apps or tools to aid parents in managing their screen time, promoting a mindful approach to technology in the presence of children and thus nurturing healthier family dynamics and supporting children’s overall development in our digital age.

## 5. Conclusions

This study identifies a positive correlation between parental phubbing and young children’s electronic media use, highlighting the complex dynamics within digitally engaged families. Our findings also reveal that parent–child conflict partially mediates this relation. A crucial aspect of the study is the moderating role of children’s emotion regulation. The data suggest that in the context of parental phubbing, children with higher levels of emotion regulation are associated with lower levels of parent–child conflict. This result implies that more developed emotion regulation skills are linked to smoother family interactions during parental phubbing, emphasizing the value of these skills in family dynamics.

## Figures and Tables

**Table 1:** Descriptive statistics and correlations of the main variables.

Variables	*M*	*SD*	1	2	3	4	5
1. Age	4.68	1.18	1				
2. Parental phubbing	2.66	0.63	0.03	1			
3. Parent–child conflict	2.16	0.69	0.08	0.45 ***	1		
4. Emotion regulation	3.04	0.47	0.09 *	0.05	−0.34 ***	1	
5. Electronic media use	2.32	0.77	0.10 *	0.38 ***	0.54 ***	−0.14 ***	1

Notes. * *p* < 0.05, *** *p* < 0.001.

## Data Availability

Data underpinning the conclusions of this research can be obtained from the corresponding author, provided the request is justified and reasonable.
